# Malignant Prostate Tissue Is Associated with Different Microbiome Gene Functions

**DOI:** 10.3390/diagnostics13020278

**Published:** 2023-01-11

**Authors:** Jae Heon Kim, Hoonhee Seo, Sukyung Kim, Asad Ul-Haq, Ho-Yeon Song, Yun Seob Song

**Affiliations:** 1 Department of Urology, Soonchunhyang University School of Medicine, Seoul 14584, Republic of Korea; 2 Department of Microbiology and Immunology, School of Medicine, Soonchunhyang University, Cheonan-si 31151, Republic of Korea; 3 Probiotics Microbiome Convergence Center, Soonchunhyang University, Asan-si 31538, Republic of Korea

**Keywords:** prostate carcinoma, microbiome, 16S rRNA

## Abstract

Specific microorganisms and changes in the constituents of the microbiome are linked with pathologies in humans, such as malignancy. Within the prostate, certain bacterial communities may locate advantageous conditions and establish themselves, thus outperforming alternative species. In this study, a comparison of malignant (MT) and benign prostate tissues (BT) or benign prostate hyperplasia (BPH) was performed in order to delineate the respective microbiomes in each sample type and to determine their pertinence to prostatic tumourigenesis. Specimens of MT (*n* = 26) and PT (*n* = 13)/BPH (*n* = 10) were acquired from patients. No variations in the make-up of the microbiome were seen when MT and PT specimens were compared. Changes in the bacterial constituents and functional genes were seen in the specimens obtained from patients with MT when contrasted against samples from those with BPH. Pelomonas was the genus with the highest abundance in MT specimens. It is proposed that dissimilar microbiome gene functions are present in the contexts of MT and PT samples.

## 1. Introduction

In humans, more than 100 trillion microbial cells live in a symbiotic relationship with their host [1]. It is well known that the presence of bacteria in certain areas of the body contribute to the functioning of the immune system, pathogenesis, and ongoing well-being. The innovation of high-throughput next-generation sequencing (NGS) techniques has led to a rise in the number of studies being conducted in order to investigate the role played by the human microbiota in relation to a number of disorders [1].

In males, prostate carcinoma is one of the most prevalent malignancies [2]. Potential contributing factors to prostate biology include infections with viruses and bacteria, as well as inflammatory triggers and environmental influences, e.g., diet and lifestyle [3,4]. Human general well-being and physiological systems are also influenced by the make-up of the microorganism population in residence, their interplay, and how they engage with the host [5,6]. These bacteria may create or contribute to an ongoing inflammatory process within neoplastic prostate tissue, although the associated mechanisms are not well delineated. Modifications in the bacterial community profiles have been identified in prostatic carcinoma that promote tumourigenesis via proinflammatory reactions or changes in the extracellular media within the prostate [7,8]. Disease-inducing pathogens believed to encourage the inflammatory process within prostatic tissue include opportunistic endogenous Enterobacteriaceae, e.g., *Escherichia coli* or *Pseudomonas* spp., and bacteria that are transmitted through sexual activity, i.e., *Neisseria gonorrhoeae*, *Chlamydia trachomatis*, and *Trichomonas vaginalis* [9]. Increased visceral inflammation has particularly been demonstrated in specimens of prostate carcinoma in the presence of *Propionibacterium acnes* [7]. Despite these data, an all-inclusive and detailed characterisation of the microbiome within pathological and normal prostate samples has not yet been published. There have been a number of publications describing the microbiome present in the male genital tract which include prokaryotic and viral DNA sequence analysis from prostate tumour samples. However, in view of the recent progress in high-throughput sequencing and bioinformatics technologies, these are no longer considered to be sufficiently in-depth or comprehensive [10,11].

Specific microorganisms and changes in the constituents of the microbiome are linked with pathologies in humans, such as neoplasia. Within the prostate, certain bacterial communities may locate advantageous conditions and establish themselves, thus outperforming alternative species. Following the delineation of the potential part played by the extracellular matrix within the malignant prostate tissue in facilitating the colonisation of certain microorganisms, it may be possible to further describe tumour development or advancement via various pathways, which may involve immune responses of the host and the constituents of the extracellular microenvironment.

In this study, a comparison of malignant (MT) and benign (BT) prostate tissues was performed in order to delineate the respective microbiomes in each sample type with the use of NGS, as well as to determine their pertinence to pathways underlying prostatic tumourigenesis. Moreover, a comparison of the microbiomes from patients with malignant MT and benign prostatic hyperplasia (BPH) was performed utilising NGS.

## 2. Materials and Methods

### 2.1. Subject Recruitment and Sample Collection

Individuals with confirmed MT (*n* = 13) and BT (*n* = 13)/BPH (*n* = 10) from the Urology Department were accepted into the study (Table S1). Tissues from prostate cancer patients allowing for both malignant (MT) and adjacent tissue benign (BT) tissue were acquired from fixing and paraffin embedding tissue in patients who underwent radical prostatectomy. MT and BT were acquired from the same patient. Tissues from BPH were acquired from fixing and paraffin embedding tissue in patients who underwent transurethral resection of prostate due to BPH.

The prostate cancer specimens had no severe concurrent disorders present, e.g., diabetes mellitus, immunocompromisation, or genetic conditions. No therapy for the prostate tumour had been administered, and no anti-microbial agents had been given for a minimum of 14 days before specimens were taken. The Ethics Committee of Soonchunhyang University Seoul Hospital gave approval for the study (2017-02-002).

### 2.2. DNA Extraction

A QIAamp DNA Mini Kit (Qiagen, Hilden, Germany) was employed for the individual extraction of metagenomic DNA; this was carried out by following the vendor’s guidelines. A NanoDrop ND-1000 spectrophotometer (ThermoFisher Scientific, Waltham, MA, USA) was used to assay the DNA quantitatively; a qualitative estimate was made utilising agarose gel electrophoresis. The DNA specimens were maintained at a temperature of −20 °C prior to subsequent assessment.

### 2.3. Illumina Sequencing and Bioinformatics Analysis of 16S rRNA Gene Amplicons

An Illumina platform was utilised for 16S rRNA gene sequencing following a technique already reported previously by our team. The primers were used for the PCR amplifications; the 16S rRNA gene V4 hypervariable section was targeted. DNA amplification was accomplished in accordance with a method described previously.

Fast length adjustment of short reads (FLASH) software was used to amalgamate pairs of reads from the initial DNA sections. Quantitative insights into microbial ecology (QIIME) software were used for sequence analysis. Allocation of sequences to operational taxonomic units (OTUs) was performed with a 97% likeness. For the individual OTUs, respective illustrative sequences were highlighted, and taxonomic data were ascribed by the RDP classifier. The illustrative sequences were divided into varying degrees of taxonomy, i.e., phylum to species, in relation to the Human Microbiome Database. A Bayesian strategy was employed with a 97% cut-off parameter.

Sampling-based OTU analysis was performed so as to appreciate the bacterial heterogeneity; this was reported as a rarefaction curve. The richness and variation in bacteria within the specimens were assessed utilising α indices, i.e., Chao 1, ACE, Simpson, Shannon, and Good’s coverage, judged at a 3% distance.

The heterogeneity of bacteria within the samples was assessed using Student’s *t*-test. Unweighted-UniFrac-distance-metrics-based PCA was carried out. Interplay between the respective bacterial populations within the specimens was appraised with the use of the R package. The constituents of the bacterial populations within the specimens were assessed using PLS-DA, nonparametric analysis of Adonis distance matrices, and ANOSIM. Distinguishing taxa amongst the two specimen cohorts at a number of strata were recognised using LEfSE (http://huttenhower.sph.harvard.edu/galaxy/ accessed on 2 December 2022) this software also allowed data to be displayed as taxonomic bar charts and cladograms. Network configurations within the specimen bacterial populations were recognised utilising the Ecological Network Analysis Pipeline, and Cytoscape was exploited to visualise them. Within the two specimen types, the functions of bacteria were forecast using the algorithm Phylogenetic Investigation of Communities by Reconstruction of Unobserved States (PICRUSt). The MeV package provided data clustering and display. The bacterial populations’ operational constituents were forecast with the use of PICRUSt in keeping with the dataset stored within the Kyoto Encyclopaedia of Genes and Genomes (KEGG). The recommendations obtained from https://github.com/picrust/picrust2/wiki accessed on 2 December 2022 were utilised for the establishment of the operational inferences of the microbiome, using PICRUSt2 together with OTUs. Analysis of variance indicated any discrepancies within the pathways.

## 3. Results

### 3.1. Overall Structure of Bacterial Communities across Samples

In the current study, 36 samples were sequenced using an Illumina system and total of 1,336,478 reads after using a pre-filter that removes low-quality reads from the raw data generated by the NGS sequencing platform. After non-specific amplicons, amplicons were not assigned to target taxa, and chimeras were removed from the QC process; the total valid reads used for data analysis were 422,258.

**Figure 1:**
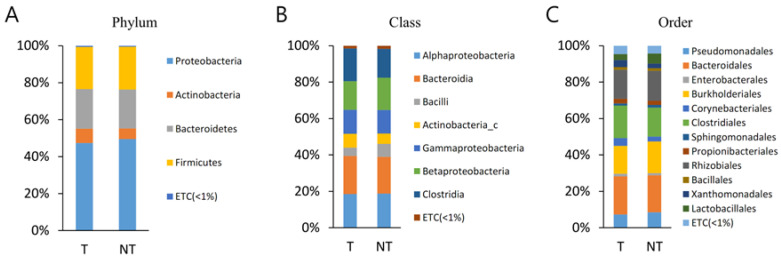

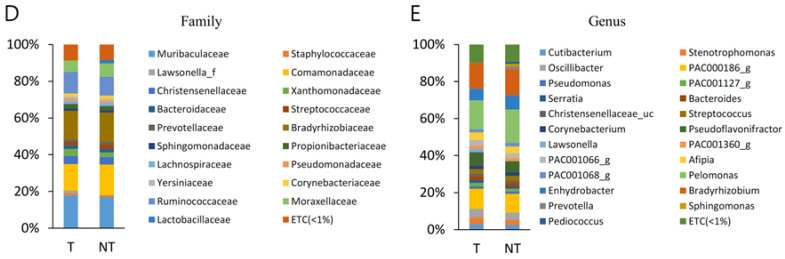
Averaged taxonomic composition in malignant prostate cancer tissues (MT) and peri-malignant prostate cancer benign prostatic tissues (BT) of prostate cancer patients. Taxonomic relative abundance was classified at the (**A**) phylum, (**B**) class, (**C**) order, (**D**) family, and (**E**) genus levels, and relative abundances less than 1% were expressed as ETC. The Wilcoxon signed-rank test was used to analyse the significance between the two. Across all ranks, none of the taxa with a relative abundance greater than 1% showed a significant difference.

There were an average of 11,729 reads per sample (ranging from 441 to 41,829 reads) with an average length 391 bp. After alignment, unique representative sequences were classified into 712 operation taxonomic units (OTUs) per sample at a 97% similarity level, from which 21 phyla, 43 classes, 95 orders, 207 families, and 806 genera were detected. Good’s estimator of coverage was 97.34%, indicating the 16S rRNA sequences identified in this study likely represent the majority of bacterial sequences present in the samples (Figure 1).

### 3.2. Bacterial Taxa (MT vs. BT)

The bacterial communities in the MT and BPH were analysed at different taxonomic levels (Table S1, Figure 1). A total of 21 phyla were identified, considering all OTUs defined. At the phyla level, Proteobacteria, Bacteroidetes, and Firmicutes were the top three most abundant phyla and together comprised 94.8% of all sequences in MT. Proteobacteria, Bacteroidetes, and Firmicutes were also the three most abundant phyla of PT and together comprised 93.4% of all sequences. The most abundant phylum was Proteobacteria, accounting for 43.2% (MT) and 45.5% (BPH) of sequences. There was no statistically different observed phyla between MT and PT.

A total of 43 classes were identified. Bacteroidia, Betaproteobacteria, Gammaproteobacteria, and Clostridia were the top four most abundant classes and together comprised 78.8% of all sequences in MT. Bacteroidia, Betaproteobacteria, Gammaproteobacteria, and Clostridia were also the top four most abundant classes of BPH and together comprised 77.5% of all sequences (Table S2). There was no statistically different observed class between MT and PT.

A total of 95 orders were identified. Bacteroidales, Burkholderiales, Clostridiales, and Pseudomonadales were the top four most abundant orders and together comprised 70.2% of all sequences in MT. Bacteroidales, Burkholderiales, Clostridiales, and Pseudomonadales were also the top four most abundant orders of BPH and together comprised 71.1% of all sequences. There was no statistical different observed order between MT and PT.

A total of 207 families were identified, considering all OTUs defined. Muribaculaceae and Comamonadaceae were the top two most abundant families and together comprised 42.9% of all sequences in MP. Muribaculaceae and Comamonadaceae were also the top four most abundant families of BPH and together comprised 43.9% of all sequences. There was no statistical different observed family between MT and PT.

A total of 806 genera were identified, considering all OTUs defined. Pelomonas and PAC000186_g were the top two most abundant genera and together comprised 30.4% of all sequences in MT. Pelomonas and PAC002482_g were also the top two most abundant genera of BPH and comprised 32.7% of all sequences. There was no statistical different observed genus between MT and PT.

### 3.3. Bacterial Taxa (MT vs. BPH)

**Figure 2:**
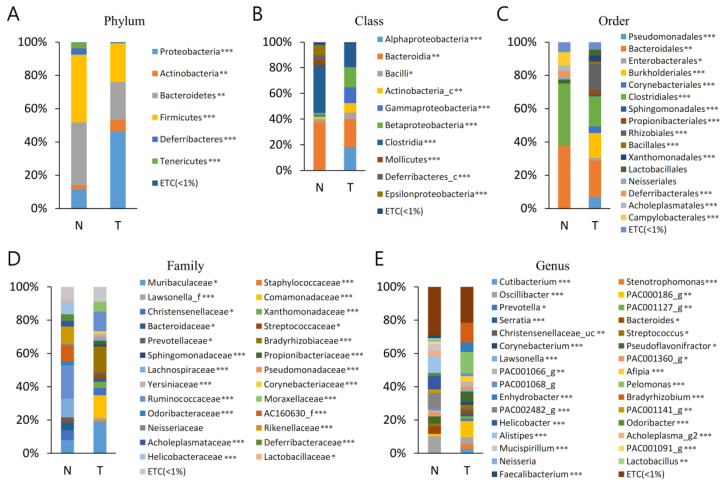
Averaged taxonomic composition in tumour regions (MT(T)) of prostate cancer patients and normal tissues (BPH (N)). Taxonomic relative abundance was classified at the (**A**) phylum, (**B**) class, (**C**) order, (**D**) family, and (**E**) genus levels, and relative abundances less than 1% are expressed as ETC. The Wilcoxon rank-sum test was used to analyse the significance between the two groups (*, *p* < 0.05; **, *p* < 0.01; ***, *p* < 0.001).

The bacterial communities in the MT and BPH were analysed at different taxonomic levels (Table S2, Figure 2). A total of 21 phyla were identified, considering all OTUs defined. At the phyla level, Proteobacteria, Bacteroidetes, and Firmicutes were the top three most abundant phyla and together comprised 93.6% of all sequences in MT. Bacteroidetes, Firmicutes, and Proteobacteria were the three most abundant phyla of BPH and together comprised 93.9% of all sequences. The most abundant phylum was Proteobacteria, accounting for 41.5% (MT), whereas Bacteroidetes accounted for 41.7% (BPH) of sequences. Proteobacteria, Actinobacteria, and Firmicutes were more abundant in T than B (*p* < 0.05). Bacteroidetes, Tenericutes, and Deferribacteres were more abundant in BPH than MT (*p* < 0.05).

A total of 43 classes were identified, considering all OTUs defined. Bacteroidia, Betaproteobacteria, Gammaproteobacteria, and Clostridia were the top four most abundant classes and together comprised 79.0% of all sequences in MT. Bacteroidia was the most abundant class of BPH and comprised 78.9% of all sequences (Table S3). Bacteroidia was the most abundant class, accounting for 31.3% (MT) and 41.5% (BPH) of sequences. Gammaproteobacteria and Betaproteobacteria were more abundant in MT than BPH (*p* < 0.05). Bacteroidia and Clostrodia were more abundant in BPH than MT (*p* < 0.05).

A total of 95 orders were identified, considering all OTUs defined. Bacteroidales, Burkholderiales, and Clostridiales were the top three most abundant orders and together comprised 62.5% of all sequences in MT. Bacteroidales and Clostridiales were the top two most abundant orders of BPH and together comprised 78.9% of all sequences. The most abundant order was Bacteroidales, accounting for 31.3% (MT) and 21.7% (BPH) of sequences. Burkholderiales was more abundant in MT than BPH (*p* < 0.05). Bacteroidales and Clostridiales were more abundant in BPH than MT (*p* < 0.05).

A total of 207 families were identified, considering all OTUs defined. Muribaculaceae and Comamonadaceae were the top two most abundant order and together comprised 43.2% of all sequences in MT. Muribaculaceae, Lachnospiraceae, Ruminococcaceae, and AC160630_f were the top four most abundant orders of BPH and together comprised 55.0% of all sequences. The most abundant family was Muribaculaceae (MT), accounting for 26.8% and Lachnospiraceae (BPH) accounting for 17.0% of sequences. Muribaculaceae and Comamonadaceae were more abundant in MT than BPH (*p* < 0.05). Lachnospiraceae, Ruminococcaceae, and AC160630_f were more abundant in BPH than MT (*p* < 0.05).

A total of 806 genera were identified, considering all OTUs defined. PAC000186_g and Pelomonas were the top two most abundant phyla and together comprised 29.9% of all sequences in MT. PAC002482_g and Helicobacter were the top two most abundant phyla of BPH, comprising 18.8% of all sequences. PAC000186_g was the most abundant genus of MT, accounting for 14.3% of sequences. PAC002482_g was the most abundant genus of BPH, accounting for 11.6% of sequences. PAC000186_g and Pelomonas were more abundant in MT than BPH (*p* < 0.05). PAC002482_g and Helicobacter were more abundant in BPH than MT (*p* < 0.05).

### 3.4. Richness and Diversity (MT vs. BT)

**Figure 3:**
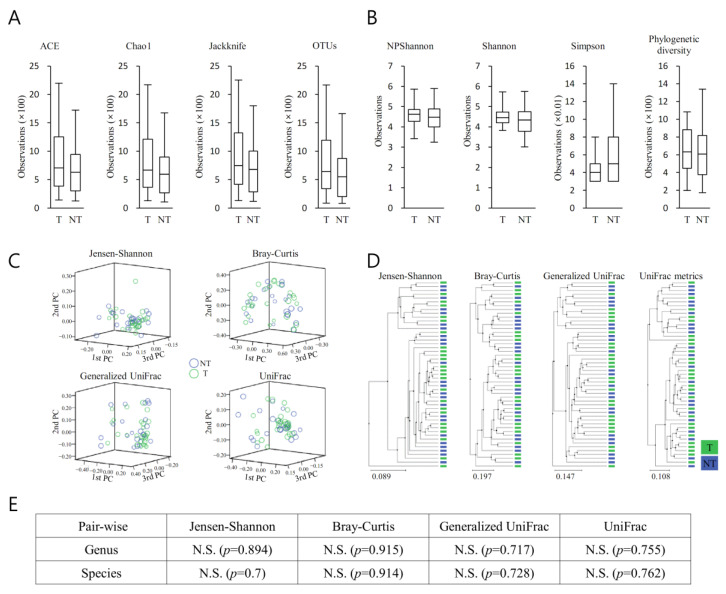
Alpha and beta diversity indices in malignant prostate cancer tissues (MT) and peri-malignant benign prostatic tissues (BT) of prostate cancer patients. (**A**) Species richness was analyzed with Ace, Chao1, Jackknife, and OTUs, (**B**) and the species diversity was analyzed with NPShannon, Shannon, Simpson, and phylogenetic diversity. The horizontal thick black band represents the median value, and the boxplot margins indicate first and third quartiles. None of the alpha diversity analysis results showed a significant difference. (**C**) Distances between communities were analysed by a principal coordinate analysis (PCoA). (**D**) Clustering using the unweighted pair group method with arithmetic mean (UPGMA) was analysed. (**E**) Beta set-significance was demonstrated by permutational multivariate analysis of variance (PERMANOVA). Beta diversity analysis was performed using Jensen–Shannon, Bray–Curtis, Generalized UniFrac, and UniFrac metrics. N.S., not significant.

The richness of the bacterial community in MT samples increased compared with that of the PT samples (Figure 3A). The diversity of the bacterial community in MT samples increased compared with that of the PT samples (Figure 3A). They demonstrated no remarkable difference between the bacterial communities between MT and BT (Figure 3B). Clustering using the unweighted pair group method with arithmetic mean (UPGMA) demonstrated that the bacterial communities in MT samples and BT samples did not cluster separately, suggesting the overall structures of the bacterial communities in the groups were not significantly different (Figure 3C). Spots representing MT samples presented no dispersed distribution patterns than those of BT samples, aligning with the no increased level of bacterial diversity found in the cancer samples. Beta set-significance was demonstrated by permutational multivariate analysis of variance (PERMANOVA) (Figure 3D). Beta diversity analysis was performed using Jensen–Shannon, Bray–Curtis, Generalized UniFrac, and UniFrac metrics, demonstrating no significant difference (Figure 3E).

### 3.5. Richness and Diversity (MP vs. BPH)

**Figure 4:**
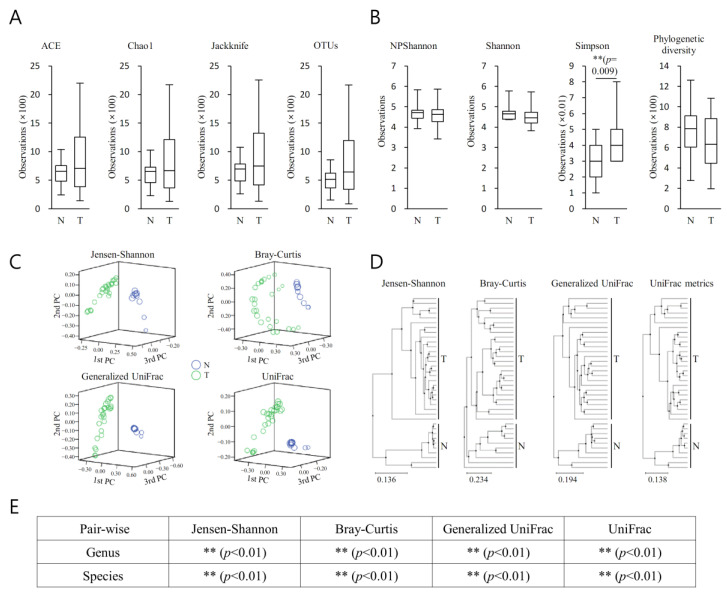
Alpha and beta diversity indices for benign prostatic tissue (BPH (N)) and tumour region in prostate cancer patients (MT(T)). (**A**) Species richness was analyzed with Ace, Chao1, Jackknife, and OTUs, (**B**) and the species diversity was analyzed with NPShannon, Shannon, Simpson, and phylogenetic diversity. The horizontal thick black band represents the median value, and the boxplot margins indicate first and third quartiles. (**C**) Distances between communities were analysed by a principal coordinate analysis (PCoA). (**D**) Clustering using the unweighted pair group method with arithmetic mean (UPGMA) was analysed. (**E**) Beta set-significance was demonstrated by permutational multivariate analysis of variance (PERMANOVA). Beta diversity analysis was performed using Jensen–Shannon, Bray–Curtis, Generalized UniFrac, and UniFrac metrics. Significance between the groups was mentioned as ** *p <* 0.01.

The richness of the bacterial community in MT samples increased compared with that of the BP samples (Figure 4A) (*p* < 0.05). The diversity of the bacterial community in BP samples increased compared with that of the MT samples (Figure 4A) (*p* < 0.05). They demonstrated remarkable differences between the bacterial communities in the groups (Figure 4C) (*p* < 0.05).

Spots representing MT samples presented more dispersed distribution patterns than those of BP samples, aligning with the increased level of bacterial diversity found in the cancer samples (*p* < 0.05). Beta set-significance was demonstrated by permutational multivariate analysis of variance (PERMANOVA) (Figure 4D). Beta diversity analysis was performed using Jensen–Shannon, Bray–Curtis, Generalized UniFrac, and UniFrac metrics, demonstrating significant differences (Figure 4E) (*p* < 0.05).

### 3.6. LEfSe (MT vs. BT)

**Figure 5:**
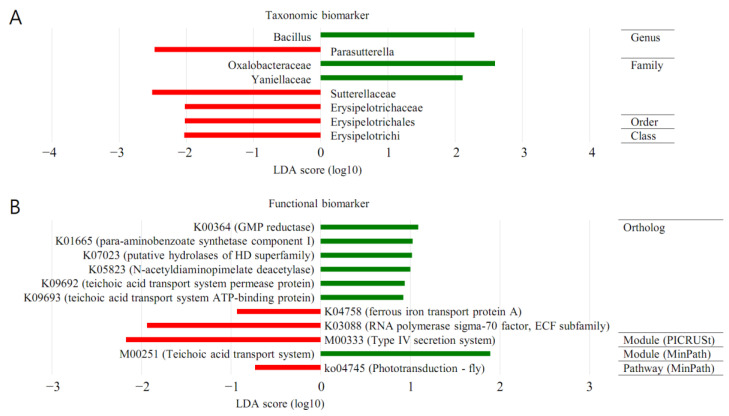
Discovery of taxonomic biomarkers and functional biomarkers in malignant prostate cancer tissues (MT) and peri-malignant prostate cancer benign prostatic tissues (BT) using LEfSe (linear discriminant analysis effect size) analysis. (**A**) In addition to taxonomic biomarkers, (**B**) functional biomarkers were analysed in ortholog, module, and pathway. For module and pathway, PICRUSt (phylogenetic investigation of communities by reconstruction of unobserved states) and MinPath (minimal set of pathways) methods were applied, but there was no significant finding when PICRUSt was applied to pathway. The KEGG (Kyoto Encyclopedia of Genes and Genomes) Database was used for functional biomarker analysis. The red area indicates more abundance in the MT group, and the green area represents vice versa.

The forest plots were generated from the LEfSe analysis, which showed the most differentially abundant taxa enriched in microbiota with green for the BT group and red for the MT group (Figure 5). At the order level, Oceanospirillales was significantly enriched in BT samples (LDA score ≥ 2). At the family level, Sutterellaceae was significantly enriched in MP samples (LDA score ≤ −2). Oxalobacteraceae was significantly enriched in BT samples (LDA score ≥ 2).

At the genus level, PAC001040_g was significantly enriched in MT samples (LDA score ≤ −2). Bacillus was significantly enriched in BT samples (LDA score ≥ 2).

### 3.7. LEfSe (MT vs. BPH)

**Figure 6:**
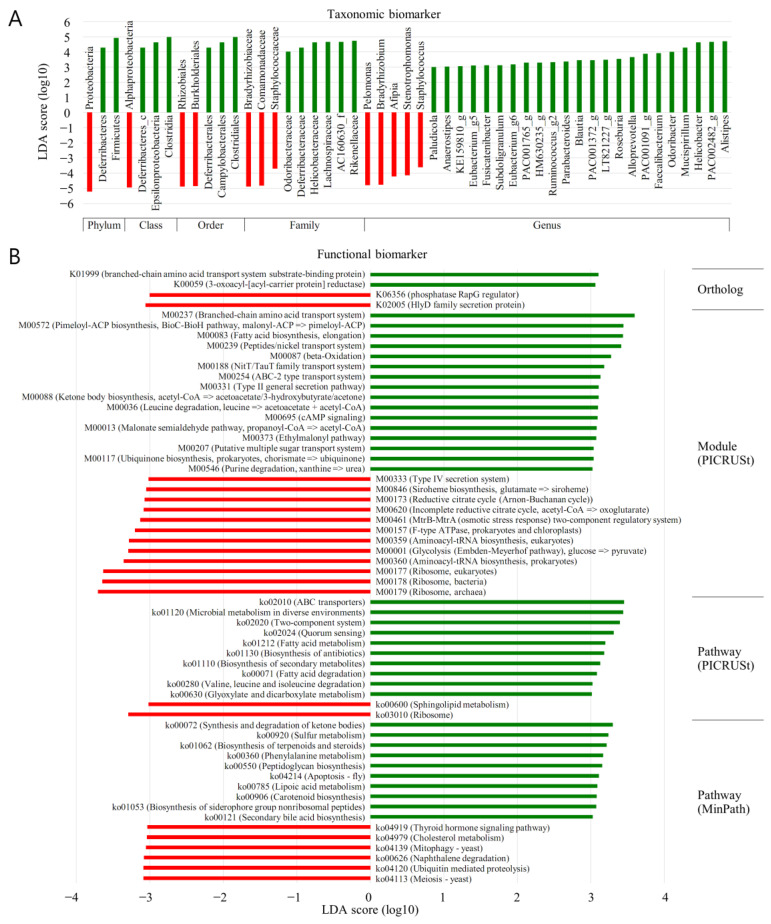
Discovery of taxonomic biomarkers and functional biomarkers in the benign prostatic tissue (N, normal) and tumour region in prostate cancer patients (T) using LEfSe (linear discriminant analysis effect size) analysis. (**A**) In addition to taxonomic biomarkers, (**B**) functional biomarkers were analysed in ortholog, module, and pathway. For module and pathway, PICRUSt (phylogenetic investigation of communities by reconstruction of unobserved states) and MinPath (minimal set of pathways) methods were applied, but there was no significant finding when MinPath was applied to module. The KEGG (Kyoto Encyclopedia of Genes and Genomes) Database was used for functional biomarker analysis. The red area indicates more abundance in the T group, and the green area indicates vice versa.

The forest plots were generated from the LEfSe analysis, which showed the most differentially abundant taxa enriched in microbiota with green for the BP group and red for the MT group (Figure 6). At the phylum level, Proteobacteria were significantly enriched in MT samples (LDA score ≤ −3). Diferribacteres and Firmicutes were significantly enriched in BP samples (LDA score ≥ 3). At the class level, alphaproteobacteria was significantly enriched in MT samples (LDA score ≤ −3). Diferribacteres_c, Epsilonproteobacteria, and clostridia were significantly enriched in BP samples (LDA score ≥ 3). At the order level, Rhozobilaes and Burkholderiales were significantly enriched in MT samples (LDA score ≤ −3). Diferribacterales, Campylobacterales, and clostridiales were significantly enriched in BPH samples (LDA score ≥ 3). At the family level, Bradyrhizobiaceae, Comamonadaceae, and Staphylococcaceae were significantly enriched in MT samples (LDA score ≤ −3). Odoribacteraceae, Diferribacteraceae, Helicobacteraceae, Lachnospiraceae, AC160630_f, and Rakenellaceae were significantly enriched in BPH samples (LDA score ≥ 3). At the genus level, Pelomonas, Bradyrhizobium, Afipia, Stenotrophomonas, and Staphylococcus were significantly enriched in MT samples (LDA score ≤ −3). Paludicola Anacrostipes, KE159810_g, Eubacterium_g5, Fusicatenibacter, Subdoligranulum, Eubacterium_g6, PAC006765_g, HM630235_g, Ruminococcus_g2, Parabacteroides, Blautia, PAC991372_g, LT821227_g, Roseburia, Alloprevotella, PAC001091_g, Faccalibacterium. Odoribacter, Mucisirillum, Helicobacter, PAC002482_g, and Alistipers were significantly enriched in BPH samples (LDA score ≥ 3).

### 3.8. PICRUSt (MT vs. BT)

The LEfSe outputs showed a series of metabolic pathways presenting significantly different distributions in each group (Figure 5). Pathways related to genetic information processing were remarkably enriched in cancer lesions. PICRUSt was performed to explore the functional profiles of microbiota associated with prostate cancer. The superpathway of the neurotriphin signaling pathway was more abundant in MP than in BPH (LDA score ≤ 0).

### 3.9. PICRUSt (MT vs. BPH)

The LEfSe outputs showed a series of metabolic pathways presenting significantly different distributions in each group (Figure 6). Pathways related to genetic information processing were remarkably enriched in cancer lesions. PICRUSt was performed to explore the functional profiles of microbiota associated with prostate cancer. The superpathway of sphingolipid metabolism, ribisome, was more abundant in MT than in BP (LDA score ≤ −3). ABC transporters; microbial metabolism in diverse environments; two component system; quorum sensing; fatty acid metabolism; biosynthesis of antibiotics; biosynthesis of secondary metabolism; fatty acid degradation; valine, leucine, and isoleucine degradation; and glyoxylate and dicarboxylate metabolism were significantly more enriched in BP samples than in MT (LDA score ≥ 3).

## 4. Discussion

Following the World Health Organisation’s categorisation of Helicobacter pylori as a carcinogen, considerable attention has been drawn to the potential association between microorganisms and the various phases of tumourigenesis [12]. Despite the fact that disease-inducing pathogens have been linked with 15.4% of tumours affecting humans [13], there are few publications which detail the part played by such organisms in the disease processes underlying prostate carcinoma.

The existence of a regional microbiome unique to prostatic tissue has been described. Numerous bacteria are present within the prostate, implying a potential pathophysiological relationship between the components making up the regional microbial community and the existence of the malignancy per se. Nevertheless, it remains unclear as to whether the varied microbiomes within prostate and peri-prostate cancerous tissues play a role [14].

In the current work, non-malignant regions of tissues were chosen from the samples obtained from the neoplastic prostate samples as BT. Comparing paired regions reduced the likelihood of intersubjective confounders, e.g., nutritional intake or lifestyle, which are recognised as affecting the make-up of the microbiome [3,4,5,6,8,14].

Within a specific disease environment within the prostate, certain colonies of bacteria may find their preferred locality in order to establish themselves and where they are able to outperform their counterparts. Enterobacteriaceae have the ability to adjust the extracellular environment through the release of enzymes, e.g., alkaline proteases and elastases, a feature implied by this pilot study to indicate relationships between malignancy and the local bacterial community [15].

The current work demonstrated that a similar taxonomic hierarchy was found between MT and BT samples. The most common three phyla were identified as Proteobacteria, Bacteroidetes, and Firmicutes; the orders of highest abundance were Bacteroidales, Burkholderiales, Clostridiales, and Pseudomonadales; the two most frequently arising families were Muribaculaceae and Comamonadaceae; and the two genera of highest abundance were Pelomonas and PAC000186_g.

We also investigated the microbial difference between MT and BPH. To date, no relationship between the bacterial community and benign and malignant prostatic conditions has been described. In the MT and BP specimens, the most frequently detected genera were PAC000186_g, which was present in 14.3% sequences, and PAC002482_g, identified in 11.6% sequences. Larger populations of PAC000186_g and Pelomonas were present in MT (*p* < 0.05); BPH contained a greater proportion of PAC002482_g and Helicobacter (*p* < 0.05). The microbiome present within BP samples was at variance with that identified within MT specimens.

Owing to the close juxtaposition of the areas of interest and the field effect, the lack of variance between the microbiome characteristics seen in MT and BT was not unexpected. However, it was possible to discern an incremental rise in the richness of a number of bacterial cohorts within each taxonomic stratum in relation to the MT and BT tissues.

The properties of the microbiome linked with the BT tissue had a greater relationship to those in the MT, suggesting a potential part played by the extracellular matrix of the prostate tumour in providing favourable conditions in which certain microorganisms could become settled. These may then influence tumourigenesis or advancement via a range of pathways, e.g., influencing the immune responses of the host and the constituents of the extracellular environs.

The anatomical location of the prostate means that it can be accessed by bacteria from both the dermatological and intestinal microbiomes; thus, the organisms within this gland may originate from both these sources. The plethora of *Propionibacterium* spp., and in particular, of *P. acnes*, is in keeping with the noted proinflammatory properties of *P. acnes* and substantiates previously observed relationships between this bacterium and prostate malignancy [8,16,17]. In the current study, no discrepancies were noted between the identified *Propionibacterium* spp. amongst the MT and BT specimens.

The increased plethora of Corynebacteriaceae, as reflected by only *Corynebacterium* spp. within the prostate abnormalities, is in keeping with the ability of such bacteria to produce a biofilm and therefore to bond with the extracellular matrix components, e.g., fibronectin, potentially in order to invade the tissue [18]. Additionally, Corynebacteriaceae are recognised aetiological factors for infections of the urinary tract or urethra [19]. In the current work, the population of *Corynebacterium* spp. was equivalent within the MT and BT areas. This research also identified that tissues from MT are linked with dissimilar microbiome components to those from BPH, being more likely to contain species of *Corynebacterium*.

An anti-tumour influence with respect to colorectal tumours has been identified in relation to *Lactobacillus* species, which appears to protect the large intestine from carcinogenesis. This effect is related to a change in the constituents of the intestinal microbiome and the release of protective factors, e.g., indole-3-lactic acid, which accelerates programmed cell death of tumour cells [20]. The quantity of *Lactobacillus* was identical in both MT and BT samples in this study. However, *Lactobacillus* were present in samples of BP, indicating a potential link between these microorganisms and benign tissue.

For each taxonomic hierarchy, an incremental alteration in the richness of a number of bacterial cohorts within the two prostatic types was seen (Figure 3). This was more evident in the MT specimens (Figure 3A). Additionally, the diversity of the bacterial populations was greater in the MT specimens (Figure 3B). PCA demonstrated clear separation between BP and MT samples (Figure 3C), with notable disparities between the bacterial populations associated with the two specimen types. Separated clustering was seen in specimens from MT and BP following clustering (UPGMA), implying that the general configurations of the microorganism populations within the MT and BPH tissues were dissimilar (Figure 4D). This difference was confirmed by beta diversity (Figure 4E). The samples from malignant prostates appeared to be linked with a heightened richness within the microbiota, whereas the specimens obtained from benign tissue demonstrated more bacterial heterogeneity.

It was demonstrated by LEfSe analysis that the orders Oceanospirillales and Sutterellaceae were abundant in BT (LDA score ≥ 2) and MP specimens (LDA score ≤ −2). From a family perspective, there was enrichment of Oxalobacteraceae in the BT specimens (LDA score ≥ 2). The genera PAC001040_g and Bacillus demonstrated abundance in the MP (LDA score ≤ −2) and BT samples (LDA score ≥ 2). Thus, following LEfSe analysis, a dissimilar microenvironment with respect to the microbiome was demonstrated in the MP samples. PICRUSt analysis showed that the neurotrophin signalling superpathway had an increased presence in the MP samples. Thus, this study demonstrated that the functional microbiome microenvironment in the MP samples was dissimilar to that identified in the BPH.

In the current work, a varied microbiota was linked with tissues obtained from prostate malignancy (MT) compared with BPH. A higher frequency of the sphingolipid metabolism superpathway, ribisome, was noted following PICRUSt analysis in malignant specimens (LDA score ≥ 3). The following operations were enhanced in tissue from benign prostates: ABC transporters; microbial metabolism in differing locales; two-component system; quorum sensing; fatty acid metabolism; antibiotic and secondary metabolism, fatty acid, and valine, leucine, and isoleucine breakdown; and glyoxylate and dicarboxylate metabolism (LDA score ≥ 3) (Figure 6B). This demonstrated variance between the functional properties of the microbiota between the two types of prostatic tissue.

From the data presented, it can be concluded that although no differences in the make-up of the bacterial populations were identified between the MT and BT specimens, alterations in bacterial gene functionality were noted. Specifically, the most frequently arising genus in the MT samples identified was Pelomonas.

## 5. Conclusions

In comparison to the specimens of BT, no differences in bacterial profiles were observed in the MT specimens, although alterations in gene functionality were recognised. It is therefore proposed that MT are related to altered gene functionality within the microbiome when contrasted against BT. When contrasted against specimens from patients with benign prostate conditions, samples from individuals with MT evidenced alterations in the make-up of the bacterial population and associated genetic functions. It is proposed that tissues from MT have a microbiome that is dissimilar to that found in BPH tissues.

## Data Availability

Due to its proprietary nature or ethical concerns, the Supporting Data cannot be made openly available. However, data could be provided for academic request after approval of the Ethics Committee.

## Supplementary Materials

The following supporting information can be downloaded at https://www.mdpi.com/article/10.3390/diagnostics13020278/s1. Table S1: Clinical characteristics of patients analyzed in this study. Table S2: Averaged taxonomic composition in malignant prostate cancer tissues (MT) and peri-malignant prostate cancer benign prostatic tissues (BT) of prostate cancer patients. Table S3: Averaged taxonomic composition in tumour regions (MT) of prostate cancer patients and benign prostate hyperplasia (BPH).
