# Supplementary material for: Malignant Prostate Tissue Is Associated with Different Microbiome Gene Functions
Source: Diagnostics (Basel). 2023 Jan 11;13(2):278. doi: 10.3390/diagnostics13020278 (PMC9858038; doi:10.3390/diagnostics13020278)
Supplement: Supplementary file 1 [file diagnostics-13-00278-s001.zip › Supplementary Table S1.pdf]

Table S1. Clinical characteristics of patients analyzed in this study

	Prostate cancer (n=13)	BPH (n=10)
Age	71.8±5.0	71.5±6.3
Survival period (day)	1780.3±46	530.5±524.4
PSA	13.2±5.5	2.9±2.6
Gleason score	7.5±0.9	
Grade group	3.1±1.3	
Pathologic T stage	T1 or T2	

PSA: prostate specific antigen.
