# Supplementary material for: Malignant Prostate Tissue Is Associated with Different Microbiome Gene Functions
Source: Diagnostics (Basel). 2023 Jan 11;13(2):278. doi: 10.3390/diagnostics13020278 (PMC9858038; doi:10.3390/diagnostics13020278)
Supplement: Supplementary file 1 [file diagnostics-13-00278-s001.zip › Supplementary Tables S2 and S3.pdf]

Table S2. – Averaged taxonomic composition in malignant prostate cancer tissues (MT) and peri-malignant prostate cancer benign prostatic tissues (BT) of prostate cancer patients.

		BT							MT						
Taxon rank	Taxon name	Ave	SD	Min	Max	Q1	Q2	Q3	Ave	SD	Min	Max	Q1	Q2	Q3
Phylum	Proteobacteria	45.5	22.4	18.5	90.0	29.9	39.1	67.5	43.2	17.2	18.9	79.5	30.4	39.1	63.9
	Actinobacteria	4.8	2.4	1.2	11.1	3.4	4.5	6.3	6.2	6.3	1.5	23.0	2.1	3.5	8.6
	Bacteroidetes	28.8	17.1	1.2	55.7	11.2	32.7	45.2	30.3	18.2	1.8	61.2	14.4	32.8	44.5
	Firmicutes	20.5	11.1	4.9	54.7	15.7	20.1	24.5	19.9	5.5	8.8	27.8	15.4	21.9	23.9
Class	Alphaproteobacteria	7.4	4.5	2.9	16.0	3.8	6.2	11.1	7.5	5.3	2.7	22.1	3.4	6.4	8.5
	Bacteroidia	28.1	17.4	0.7	55.3	10.2	32.2	44.9	29.8	18.4	1.0	60.7	13.6	32.3	44.4
	Bacilli	8.9	10.5	2.4	48.2	3.1	4.9	10.1	6.3	4.1	1.1	15.8	2.7	5.8	9.3
	Actinobacteria_c	4.8	2.4	1.2	11.1	3.4	4.5	6.3	6.2	6.3	1.5	23.0	2.1	3.5	8.5
	Gammaproteobacteria	16.8	8.6	6.7	44.5	10.5	15.6	24.4	17.0	9.3	5.4	50.3	10.8	16.5	21.8
	Betaproteobacteria	21.2	15.7	5.6	56.6	9.2	13.2	31.4	18.7	10.1	5.9	45.1	10.1	17.5	23.1
	Clostridia	11.4	6.5	0.5	21.6	5.2	12.6	16.8	13.3	7.1	1.0	22.6	7.6	15.6	18.9
Order	Pseudomonadales	10.7	8.2	3.4	42.5	5.9	8.9	12.2	9.2	4.8	2.1	17.0	4.6	10.2	13.4
	Bacteroidales	28.1	17.4	0.7	55.3	10.2	32.2	44.9	29.8	18.4	1.0	60.7	13.6	32.3	44.4
	Enterobacterales	2.4	2.1	0.4	9.3	1.2	1.5	2.9	3.0	4.6	0.4	20.2	0.8	1.3	2.5
	Burkholderiales	20.9	15.8	5.2	56.3	9.1	13.1	31.0	18.1	9.9	5.3	44.7	9.9	17.1	22.8
	Corynebacteriales	2.3	1.5	0.6	6.8	1.4	1.7	3.1	3.4	4.7	0.6	18.1	0.8	1.4	4.9
	Clostridiales	11.4	6.5	0.5	21.5	5.0	12.6	16.7	13.3	7.1	1.0	22.4	7.6	15.6	18.9
	Sphingomonadales	1.1	1.6	0.1	7.6	0.3	0.6	0.9	0.9	1.6	0.1	6.4	0.3	0.4	0.6
	Propionibacteriales	2.0	0.8	0.5	3.0	1.1	2.0	2.8	2.2	2.0	0.5	9.4	0.9	1.6	2.9
	Rhizobiales	5.8	4.0	2.0	14.7	2.6	4.7	6.7	5.4	3.4	2.2	13.7	2.4	4.6	6.8
	Bacillales	2.2	1.5	0.4	7.8	1.1	2.1	2.4	2.2	1.9	0.2	6.9	0.7	1.9	2.3
	Xanthomonadales	2.9	3.1	0.3	10.6	1.2	1.6	2.8	4.2	5.2	0.3	24.5	0.8	2.4	6.4
	Lactobacillales	6.7	9.7	1.4	46.0	2.0	3.4	7.3	4.1	2.5	0.9	9.0	1.9	3.7	6.3
Family	Muribaculaceae	23.8	15.1	0.3	45.1	9.1	23.1	39.7	25.6	16.1	0.4	52.7	12.2	27.9	38.1
	Staphylococcaceae	1.6	1.0	0.4	5.1	0.9	1.4	2.0	1.8	1.7	0.2	6.2	0.6	1.4	1.8
	Lawsonella_f	0.4	0.3	0.0	1.6	0.2	0.3	0.4	1.0	2.7	0.0	13.4	0.2	0.4	0.7
	Comamonadaceae	20.1	15.8	4.4	55.2	8.7	12.5	30.3	17.3	9.9	4.6	44.2	9.3	15.9	21.5
	Christensenellaceae	3.1	2.0	0.0	6.8	1.2	3.3	4.6	3.5	2.2	0.1	6.7	0.9	3.9	5.1
	Xanthomonadaceae	2.9	3.1	0.3	10.6	1.2	1.6	2.8	4.2	5.2	0.3	24.5	0.8	2.4	6.4
	Bacteroidaceae	2.7	2.0	0.0	8.1	1.1	2.8	4.2	3.0	2.1	0.2	7.0	0.7	2.8	4.6
	StreBTococcaceae	3.1	4.1	0.6	19.9	1.0	2.1	3.1	2.7	2.1	0.5	7.7	0.8	1.8	4.1
	Prevotellaceae	1.4	1.6	0.1	6.4	0.3	0.9	1.8	1.1	1.2	0.1	5.4	0.5	0.7	1.2
	Bradyrhizobiaceae	5.1	4.0	1.3	14.5	2.4	3.6	5.5	5.0	3.5	1.4	13.5	2.1	4.4	6.6
	Sphingomonadaceae	1.1	1.6	0.1	7.6	0.3	0.5	0.8	0.9	1.6	0.1	6.3	0.2	0.4	0.6
	Propionibacteriaceae	2.0	0.8	0.5	3.0	1.1	2.0	2.8	2.2	2.0	0.5	9.4	0.9	1.5	2.9
	Lachnospiraceae	2.1	1.1	0.2	4.5	1.2	2.1	2.9	2.4	1.2	0.0	4.2	1.7	2.7	3.3
	Pseudomonadaceae	1.6	2.1	0.3	10.2	0.8	1.0	1.6	1.8	2.5	0.2	12.0	0.7	1.3	1.6
	Yersiniaceae	2.2	2.1	0.3	9.1	1.2	1.5	2.5	2.8	4.5	0.3	19.7	0.5	1.2	2.1
	Corynebacteriaceae	1.8	1.4	0.4	5.8	0.9	1.2	2.6	1.7	1.8	0.3	6.6	0.5	0.8	2.1
	Ruminococcaceae	6.1	3.5	0.2	11.4	2.5	6.6	9.3	7.3	3.9	0.7	12.8	3.7	8.4	10.2
	Moraxellaceae	9.1	8.4	1.6	42.0	3.8	7.2	10.9	7.4	4.8	1.4	15.5	2.7	7.7	11.5
	Lactobacillaceae	2.3	7.7	0.1	37.2	0.2	0.4	1.0	0.6	0.6	0.0	2.5	0.2	0.5	0.7

Genus	Cutibacterium	2.0	0.8	0.5	3.0	1.1	2.0	2.8	2.2	2.0	0.5	9.4	0.9	1.5	2.9
	Stenotrophomonas	2.8	3.1	0.3	10.6	1.2	1.6	2.8	3.8	5.2	0.2	24.4	0.6	2.1	5.3
	Oscillibacter	2.0	1.4	0.0	5.3	0.7	2.0	2.9	2.3	1.3	0.2	3.9	0.7	2.4	3.6
	PAC000186_g	12.9	8.5	0.1	29.8	3.8	14.2	18.6	13.9	8.7	0.2	28.8	4.3	15.8	21.1
	Prevotella	1.3	1.6	0.0	6.0	0.3	0.8	1.8	1.1	1.1	0.1	5.0	0.5	0.7	1.2
	PAC001127_g	2.0	1.1	0.0	3.6	1.1	2.3	2.9	2.4	1.5	0.0	4.8	0.7	3.0	3.4
	Serratia	2.1	2.1	0.3	9.0	1.1	1.5	2.4	2.7	4.5	0.3	19.6	0.5	1.2	2.1
	Bacteroides	2.7	2.0	0.0	8.1	1.1	2.8	4.2	3.0	2.1	0.2	7.0	0.7	2.8	4.6
	Christensenellaceae_uc	0.8	0.7	0.0	2.6	0.1	0.7	1.2	0.9	0.6	0.0	2.2	0.1	1.0	1.2
	Streptococcus	2.9	4.0	0.6	19.9	1.0	1.8	2.8	2.6	2.1	0.5	7.6	0.8	1.8	3.9
	Corynebacterium	1.8	1.4	0.4	5.7	0.9	1.1	2.4	1.6	1.7	0.3	6.4	0.5	0.8	2.1
	Pseudoflavonifractor	3.0	1.9	0.1	6.1	1.2	3.1	4.6	3.7	2.3	0.1	8.4	1.4	4.4	5.1
	Lawsonella	0.4	0.3	0.0	1.6	0.2	0.3	0.4	1.0	2.7	0.0	13.4	0.2	0.4	0.7
	PAC001360_g	1.2	0.8	0.0	2.7	0.5	1.4	1.9	1.4	1.0	0.0	3.1	0.5	1.8	2.2
	PAC001066_g	3.0	2.1	0.0	8.4	1.1	3.3	4.5	3.7	2.3	0.1	8.3	1.2	4.2	5.3
	Afipia	1.0	0.9	0.0	3.2	0.5	0.7	1.3	1.1	0.9	0.2	3.5	0.4	0.7	1.3
	PAC001068_g	2.2	2.9	0.0	10.0	0.2	1.2	2.4	2.1	3.0	0.0	11.6	0.6	1.4	2.1
	Pelomonas	19.8	15.8	4.4	54.9	8.6	12.0	29.9	16.5	10.0	4.5	44.2	9.0	14.2	20.4
	Enhydrobacter	8.1	8.4	1.1	41.0	2.9	7.0	9.5	6.6	5.0	0.8	14.8	1.4	6.9	11.1
	Bradyrhizobium	4.1	3.1	1.3	11.2	1.8	2.7	4.3	3.9	2.7	1.1	10.7	1.6	3.2	5.1
	Pseudomonas	1.5	2.1	0.3	10.1	0.5	0.9	1.6	1.8	2.5	0.2	11.8	0.7	1.2	1.5
	Sphingomonas	1.0	1.6	0.1	7.6	0.2	0.5	0.8	0.8	1.5	0.1	6.2	0.2	0.3	0.6
	Pediococcus	1.2	4.8	0.0	23.3	0.0	0.1	0.1	0.1	0.3	0.0	1.3	0.0	0.1	0.1

Relative abundances less than 1 % were expressed as ETC. Wilcoxon signed-rank test was used to analyze the significance between the two groups. Ave, average; SD, standard deviation; Min, minimum; Max, maximum; Q1, first quartile; Q2: median; Q3, third quartile.

Table S3. – Averaged taxonomic composition in tumor regions (MT) of prostate cancer patients and benign prostate hyperplasia (BPH).

		MT							BPH						
Taxon rank	Taxon name	Ave	SD	Min	Max	Q1	Q2	Q3	Ave	SD	Min	Max	Q1	Q2	Q3
Phylum	Proteobacteria***	41.5	16.9	18.9	79.5	30.0	37.9	58.0	11.3	3.4	4.8	18.2	9.3	12.0	12.3
	Actinobacteria**	6.0	6.0	1.5	23.0	2.2	3.5	6.7	1.5	1.8	0.3	5.3	0.4	0.6	2.1
	Bacteroidetes**	31.8	17.8	1.8	61.2	14.5	33.5	44.5	41.7	8.1	25.8	56.5	37.6	42.1	46.5
	Firmicutes***	20.3	5.4	8.8	27.8	15.5	22.2	23.9	40.9	5.3	34.4	49.1	35.9	40.4	44.7
	Deferribacteres***	0.0	0.1	0.0	0.1	0.0	0.0	0.1	2.3	1.1	0.1	3.4	1.9	2.7	3.1
	Tenericutes***	0.1	0.1	0.0	0.2	0.0	0.1	0.1	2.1	1.2	0.0	3.1	0.5	2.8	3.0
Class	Alphaproteobacteria***	7.3	5.0	2.7	22.1	4.0	6.2	8.3	0.2	0.2	0.0	0.6	0.0	0.1	0.2
	Bacteroidia**	31.3	18.1	1.0	60.7	13.8	32.9	44.4	41.5	8.3	25.1	56.5	37.5	42.0	46.4
	Bacilli*	6.6	4.5	1.1	18.2	3.4	5.5	9.5	3.3	3.7	0.1	12.0	0.6	2.1	5.0
	Actinobacteria_c**	6.0	5.9	1.5	23.0	2.2	3.5	6.7	1.2	1.6	0.2	5.0	0.3	0.5	1.7
	Gammaproteobacteria***	16.0	9.3	4.1	50.3	10.3	13.9	21.1	2.2	3.1	0.3	8.3	0.5	0.6	3.2
	Betaproteobacteria***	18.2	9.7	5.9	45.1	10.6	16.7	22.4	1.5	3.1	0.1	10.0	0.1	0.2	1.4
	Clostridia***	13.5	6.9	1.0	22.6	8.1	15.2	18.9	37.4	3.3	33.8	42.6	34.7	36.1	41.5
	Mollicutes***	0.1	0.1	0.0	0.2	0.0	0.1	0.1	2.1	1.2	0.0	3.1	0.5	2.8	3.0
	Deferribacteres_c***	0.0	0.1	0.0	0.1	0.0	0.0	0.1	2.3	1.1	0.1	3.4	1.9	2.7	3.1
	Epsilonproteobacteria***	0.0	0.0	0.0	0.2	0.0	0.0	0.0	7.2	4.0	0.1	11.4	3.5	8.3	9.9
Order	Pseudomonadales***	8.8	5.0	1.8	17.0	3.9	9.9	13.0	0.3	0.4	0.0	1.4	0.1	0.2	0.4
	Bacteroidales**	31.3	18.1	1.0	60.7	13.8	32.9	44.4	41.5	8.3	25.1	56.5	37.5	42.0	46.4
	Enterobacterales*	2.8	4.4	0.3	20.2	0.8	1.3	2.4	1.5	2.1	0.3	6.2	0.4	0.5	2.0
	Burkholderiales***	17.7	9.5	5.3	44.7	10.4	16.5	22.2	0.4	0.7	0.1	2.1	0.1	0.1	0.5
	Corynebacteriales***	3.1	4.5	0.6	18.1	0.8	1.4	3.0	0.2	0.3	0.0	0.7	0.0	0.1	0.3
	Clostridiales***	13.5	6.9	1.0	22.4	8.1	15.2	18.9	37.4	3.3	33.8	42.6	34.7	36.1	41.5
	Sphingomonadales***	0.8	1.5	0.1	6.4	0.3	0.4	0.6	0.0	0.1	0.0	0.3	0.0	0.0	0.0
	Propionibacteriales***	2.2	1.9	0.5	9.4	1.0	1.7	2.9	0.3	0.3	0.0	1.0	0.1	0.2	0.4
	Rhizobiales***	5.3	3.2	2.2	13.7	2.6	4.7	6.7	0.0	0.1	0.0	0.2	0.0	0.0	0.1
	Bacillales***	2.1	1.8	0.2	6.9	0.8	2.0	2.3	0.1	0.1	0.0	0.3	0.0	0.1	0.3
	Xanthomonadales***	3.9	5.0	0.3	24.5	0.8	2.3	5.7	0.1	0.2	0.0	0.5	0.0	0.0	0.1
	Lactobacillales	4.5	3.3	0.9	15.8	2.4	3.4	6.3	3.2	3.6	0.1	11.7	0.6	2.0	4.8
	Neisseriales	0.5	0.6	0.0	2.4	0.1	0.4	0.6	1.0	2.4	0.0	7.8	0.0	0.1	0.9
	Deferribacterales***	0.0	0.1	0.0	0.1	0.0	0.0	0.1	2.3	1.1	0.1	3.4	1.9	2.7	3.1
	Acholeplasmatales***	0.0	0.1	0.0	0.2	0.0	0.0	0.1	2.1	1.2	0.0	3.1	0.5	2.8	3.0
	Campylobacterales***	0.0	0.0	0.0	0.2	0.0	0.0	0.0	7.2	4.0	0.1	11.4	3.5	8.3	9.9
Family	Muribaculaceae*	26.8	15.8	0.4	52.7	12.3	27.9	38.5	11.3	4.9	1.6	15.2	8.2	13.5	14.9
	Staphylococcaceae***	1.7	1.6	0.2	6.2	0.6	1.4	1.8	0.0	0.0	0.0	0.1	0.0	0.0	0.0
	Lawsonella_f***	0.9	2.6	0.0	13.4	0.1	0.4	0.7	0.0	0.0	0.0	0.1	0.0	0.0	0.1
	Comamonadaceae***	16.4	9.7	4.6	44.2	9.2	14.2	20.7	0.2	0.3	0.0	0.8	0.0	0.1	0.2
	Christensenellaceae*	3.6	2.1	0.1	6.7	1.7	3.9	5.1	5.1	2.4	0.2	7.2	4.1	5.9	6.6
	Xanthomonadaceae***	3.9	5.0	0.3	24.5	0.8	2.3	5.7	0.1	0.2	0.0	0.5	0.0	0.0	0.1
	Bacteroidaceae*	3.0	2.0	0.2	7.0	1.5	2.9	4.5	6.4	5.5	2.4	18.1	3.1	4.2	8.4
	Streptococcaceae*	3.1	3.1	0.5	14.7	1.3	1.9	4.1	1.3	2.4	0.0	7.6	0.1	0.1	2.2
	Prevotellaceae*	1.3	1.4	0.1	5.4	0.5	0.8	1.8	2.5	1.1	0.4	4.1	2.0	2.4	3.4
	Bradyrhizobiaceae***	4.9	3.3	1.4	13.5	2.2	4.5	6.2	0.0	0.0	0.0	0.1	0.0	0.0	0.0
	Sphingomonadaceae***	0.8	1.5	0.1	6.3	0.2	0.4	0.6	0.0	0.1	0.0	0.3	0.0	0.0	0.0
	Propionibacteriaceae***	2.2	1.9	0.5	9.4	0.9	1.6	2.9	0.3	0.3	0.0	1.0	0.1	0.2	0.4
	Lachnospiraceae***	2.4	1.2	0.0	4.2	1.7	2.7	3.3	17.0	2.3	13.8	21.0	15.4	16.4	19.0
	Pseudomonadaceae***	1.7	2.4	0.2	12.0	0.6	1.0	1.5	0.0	0.0	0.0	0.1	0.0	0.0	0.0
	Yersiniaceae***	2.5	4.3	0.3	19.7	0.5	1.2	2.0	0.1	0.3	0.0	1.1	0.0	0.0	0.0
	Corynebacteriaceae***	1.6	1.7	0.3	6.6	0.5	0.8	2.0	0.1	0.2	0.0	0.6	0.0	0.1	0.1
	Ruminococcaceae***	7.4	3.7	0.7	12.8	4.7	8.4	10.2	15.0	1.9	12.5	18.4	13.5	14.7	16.6
	Moraxellaceae***	7.1	4.9	1.4	15.5	2.1	7.3	11.6	0.3	0.4	0.0	1.3	0.0	0.1	0.4
	Odoribacteraceae***	0.0	0.0	0.0	0.0	0.0	0.0	0.0	2.3	1.0	0.5	3.6	1.2	2.6	3.2
	AC160630_f***	0.0	0.0	0.0	0.0	0.0	0.0	0.0	11.7	8.6	1.2	31.9	6.2	10.9	14.9
	Neisseriaceae	0.5	0.6	0.0	2.4	0.1	0.4	0.6	1.0	2.4	0.0	7.8	0.0	0.1	0.9
	Rikenellaceae***	0.0	0.0	0.0	0.1	0.0	0.0	0.0	6.2	2.5	1.0	8.0	5.1	7.4	7.8
	Acholeplasmataceae***	0.0	0.1	0.0	0.2	0.0	0.0	0.1	2.1	1.2	0.0	3.1	0.5	2.8	3.0
	Deferribacteraceae***	0.0	0.1	0.0	0.1	0.0	0.0	0.1	2.3	1.1	0.1	3.4	1.9	2.7	3.1
	Helicobacteraceae***	0.0	0.0	0.0	0.0	0.0	0.0	0.0	7.2	4.0	0.0	11.4	3.5	8.3	9.9
	Lactobacillaceae*	0.5	0.6	0.0	2.5	0.2	0.5	0.7	1.4	1.2	0.1	3.8	0.5	1.1	2.2
Genus	Cutibacterium***	2.2	1.9	0.5	9.4	0.9	1.6	2.9	0.2	0.3	0.0	1.0	0.1	0.2	0.3
	Stenotrophomonas***	3.5	5.0	0.2	24.4	0.6	2.0	4.6	0.0	0.0	0.0	0.1	0.0	0.0	0.0
	Oscillibacter***	2.4	1.3	0.2	3.9	1.2	2.6	3.5	6.0	2.3	1.8	8.7	5.3	6.8	7.1
	PAC000186_g**	14.3	8.2	0.2	28.8	7.2	16.0	19.4	1.8	0.7	0.4	2.4	1.3	2.0	2.3
	Prevotella*	1.3	1.3	0.1	5.0	0.5	0.8	1.8	0.6	0.5	0.1	2.0	0.3	0.4	0.6
	PAC001127_g**	2.4	1.4	0.0	4.8	0.7	2.9	3.3	0.3	0.2	0.0	0.5	0.3	0.4	0.4
	Serratia***	2.5	4.2	0.2	19.6	0.5	1.2	2.0	0.1	0.3	0.0	1.1	0.0	0.0	0.0
	Bacteroides*	3.0	2.0	0.2	7.0	1.5	2.9	4.5	6.4	5.5	2.4	18.1	3.1	4.2	8.4
	Christensenellaceae_uc**	1.0	0.6	0.0	2.2	0.3	1.0	1.3	0.3	0.2	0.0	0.6	0.1	0.3	0.5
	Streptococcus*	3.0	3.1	0.5	14.7	1.3	1.9	4.0	1.3	2.4	0.0	7.5	0.1	0.1	2.2
	Corynebacterium***	1.6	1.6	0.3	6.4	0.5	0.8	1.9	0.1	0.2	0.0	0.6	0.0	0.1	0.1

	Pseudoflavonifractor*	3.7	2.1	0.1	8.4	1.6	4.3	5.0	2.6	1.1	0.6	3.7	1.6	3.0	3.4
	Lawsonella***	0.9	2.6	0.0	13.4	0.1	0.4	0.7	0.0	0.0	0.0	0.1	0.0	0.0	0.1
	PAC001360_g*	1.4	1.0	0.0	3.1	0.6	1.6	2.2	2.2	1.1	0.1	3.8	1.8	2.5	2.7
	PAC001066_g**	3.8	2.2	0.1	8.3	1.7	4.2	5.1	1.5	0.8	0.0	2.2	1.1	1.8	2.1
	Afipia***	1.1	0.8	0.2	3.5	0.4	0.9	1.2	0.0	0.0	0.0	0.0	0.0	0.0	0.0
	PAC001068_g	2.5	3.2	0.0	11.6	0.6	1.4	2.6	1.6	0.8	0.1	2.5	0.7	2.0	2.2
	Pelomonas***	15.6	9.7	4.5	44.2	9.0	12.2	20.0	0.1	0.2	0.0	0.5	0.0	0.1	0.1
	Enhydrobacter***	6.4	5.0	0.8	14.8	1.4	6.1	11.1	0.2	0.3	0.0	1.1	0.0	0.1	0.3
	Bradyrhizobium***	3.9	2.5	1.1	10.7	1.8	3.4	5.0	0.0	0.0	0.0	0.0	0.0	0.0	0.0
	PAC002482_g***	0.0	0.0	0.0	0.0	0.0	0.0	0.0	11.6	8.7	0.5	31.9	6.1	10.8	14.9
	PAC001141_g**	0.4	0.3	0.0	0.9	0.1	0.6	0.7	1.7	1.0	0.0	2.8	0.5	2.1	2.6
	Helicobacter***	0.0	0.0	0.0	0.0	0.0	0.0	0.0	7.2	4.0	0.0	11.4	3.5	8.3	9.9
	Odoribacter***	0.0	0.0	0.0	0.0	0.0	0.0	0.0	2.2	1.1	0.3	3.6	1.2	2.6	3.2
	Alistipes***	0.0	0.0	0.0	0.1	0.0	0.0	0.0	5.9	2.3	1.0	7.6	4.8	6.8	7.3
	Acholeplasma_g2***	0.0	0.1	0.0	0.2	0.0	0.0	0.1	2.1	1.2	0.0	3.1	0.5	2.8	3.0
	Mucispirillum***	0.0	0.1	0.0	0.1	0.0	0.0	0.1	2.3	1.1	0.1	3.4	1.9	2.7	3.1
	PAC001091_g***	0.0	0.1	0.0	0.2	0.0	0.0	0.1	2.7	1.3	0.4	4.1	2.0	2.8	3.8
	Neisseria	0.4	0.4	0.0	2.0	0.1	0.2	0.4	1.0	2.3	0.0	7.5	0.0	0.1	0.9
	Lactobacillus**	0.4	0.4	0.0	1.3	0.2	0.3	0.6	1.4	1.2	0.1	3.8	0.5	1.1	2.2
	Faecalibacterium***	0.0	0.0	0.0	0.1	0.0	0.0	0.0	2.6	3.4	0.5	9.1	0.8	1.0	3.8

Relative abundances less than 1 % were expressed as ETC. Wilcoxon rank-sum test was used to analyze the significance between the two groups (*, $p < 0.05$; **, $p < 0.01$; ***, $p < 0.001$). Ave, average; SD, standard deviation; Min, minimum; Max, maximum; Q1, first quartile; Q2: median; Q3, third quartile.
